# Hundred most cited articles in perioperative neurocognitive disorder: a bibliometric analysis

**DOI:** 10.1186/s12871-021-01408-4

**Published:** 2021-07-02

**Authors:** Xinning Mi, Xiaoxiao Wang, Ning Yang, Yongzheng Han, Yue Li, Taotao Liu, Dengyang Han, Yi Yuan, Yiyun Cao, Chengmei Shi, Xiangyang Guo, Yang Zhou, Zhengqian Li

**Affiliations:** 1 Department of Anesthesiology, Peking University Third Hospital, No.49 North Garden Road, Haidian District, 100191 Beijing, China; 2 Research Center of Clinical Epidemiology, Peking University Third Hospital, 100191 Beijing, China; 3 Department of Anesthesiology, Beijing Jishuitan Hospital, 100035 Beijing, China; 4 Department of Anesthesiology, Shanghai Sixth People’s Hospital East Affiliated with Shanghai University of Medicine and Health Sciences, 200233 Shanghai, China

**Keywords:** Perioperative neurocognitive disorder, Postoperative cognitive dysfunction, Postoperative delirium, Bibliometric analysis

## Abstract

**Background:**

In line with aging populations and increased application of anesthesia and surgery, perioperative neurocognitive disorder (PND) has received growing attention worldwide. Considerable researches into PND are being conducted; however, the quantity and quality of such researches have not been reported. Through a retrospective bibliometric analysis, this study aims to identify and characterize the top 100 cited publications on PND.

**Methods:**

We searched the Web of Science database to find the top 100 cited articles focusing on PND. We collected bibliographic information, including year of publication, country of origin, article type, published journal, citation count, and authorship. To determine changes with time, we compared older and newest articles.

**Results:**

The top 100 cited articles were published between 1955 and 2016; the number of citations ranged from 111 to 1248. The United States had the most published papers; clinical trial was the most common article type. The specialty journals of *Anesthesiology* and *Anesthesia & Analgesia* were the two most cited journals. Newest articles had a comparable number of citations to older articles, but the former had higher annual citation rates, greater funding disclosures, more focus on basic research, and more open access publications.

**Conclusions:**

This study provides a comprehensive overview of the most cited articles and highlights the increasing attention on PND. High-quality clinical trials with a greater journal impact factor receive more citations. However, there has been a growth in the number of basic science studies as an area of research with respect to the pathogenesis of PND.

## Background

Formerly known as postoperative delirium (POD) and postoperative cognitive dysfunction (POCD), perioperative neurocognitive disorder (PND) is an overarching concept for identifying cognitive impairment during the preoperative or postoperative period [1]. PND is one of the most common perioperative complications observed in older individuals who receive surgery under general or regional anesthesia. PND is evidenced as disorders in executive function, memory, and other cognitive aspects for a period ranging from hours to months. This clinical syndrome was first reported in 1955 by Bedford [2]. Since then, many studies have examined PND from multiple perspectives, including risk factors, prevention, treatment, probable mechanisms, and with a focus on humans, rodents, and cells. PND is currently one of the most frequently studied areas in perioperative medicine.

Bibliometric studies are important tools in evaluating research performance and identifying influential papers in a particular field. One investigation conducted a bibliometric analysis of publications on POCD between 2000 and 2019; it identified publication trends and hot spots in POCD research over the 20-year period [3]. However, a bibliometric analysis of high quality, top-cited papers on PND has yet to be carried out. By comparing changes in the citation trends of published papers, it is possible to better understand the current research situation and determine the direction for future efforts. The present study aimed to assess the 100 most cited papers on PND using bibliometric analysis to identify the nature, content, and their shifts with time.

## Materials and methods

### Search strategy

We examined publications focusing on PND from 1955 to 2020 using the Web of Science database. The key words we applied were “postoperative cognitive dysfunction,” “postoperative delirium,” “perioperative neurocognitive disorder,” “surgery,” “surgical,” “anesthetic,” and “cognitive dysfunction” combined with AND and OR Boolean operators. We limited the search to English-language publications. We conducted the article search using the Science Citation Index Expanded database of the Web of Science Core Collection to obtain the 100 most cited papers that focused on PND. We collected the following bibliometric information: year of publication; country of origin; article type (basic research, clinical randomized controlled trial, clinical cohort study, clinical case-control study, clinical case series, narrative review or expert opinion, and systematic review or meta-analysis); published journal; citation count; and authorship. We applied no exclusion criteria. Further, we sorted the papers by date of publication; to evaluate the research characteristic shifts over time, we divided into them into 50 older articles (before February 2008) and 50 newest articles (after June 2008).

### Statistical analysis

We performed statistical analysis using SPSS software (version 21.0; IBM Corp., Armonk, NY, USA). The data were expressed as the mean (range) or number (%). We analyzed categorical variables using a χ2 test and continuous variables with an independent-sample t test. We calculated correlation coefficients (r) and P values using Spearman’s test. A P value of < 0.05 was considered statistically significant.

## Results

### Year and country of publication

**Fig. 1:**
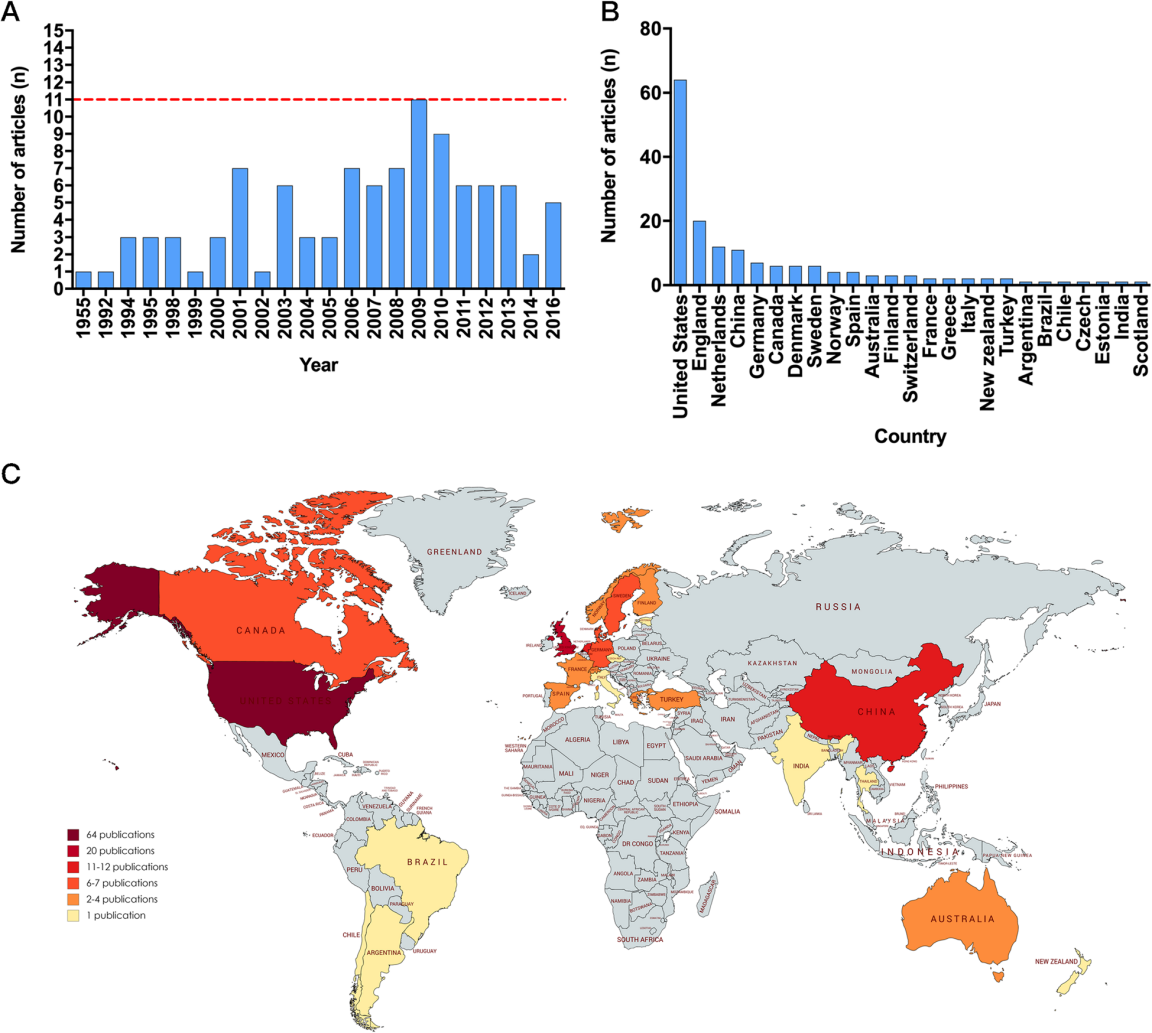
*** A***, Publication year of the 100 most cited articles; ***B-C***, Contribution of countries and regions published the most highly cited papers

The publication year of the 100 most cited papers on PND ranged from 1955 to 2016, with the majority of papers being published in the 2000 s (*n* = 88). Overall, the number of published papers showed a steady growth trend. Notably, from 2006 to 2013, the number of high-citation papers was over six per year. Most papers were published in 2009 (*n* = 11; Fig. 1 A). The authors from the United States published the most papers (*n* = 63); it was followed by England (*n* = 20), the Netherlands (n = 12), and China (*n* = 11). The authors from Germany contributed seven articles; it was followed by Canada, Denmark, and Sweden, which each had six (Fig. 1B). In all, 25 countries accounted for the 100 most cited articles that focused on PND (Fig. 1 C).

### Study characteristics

**Fig. 2:**
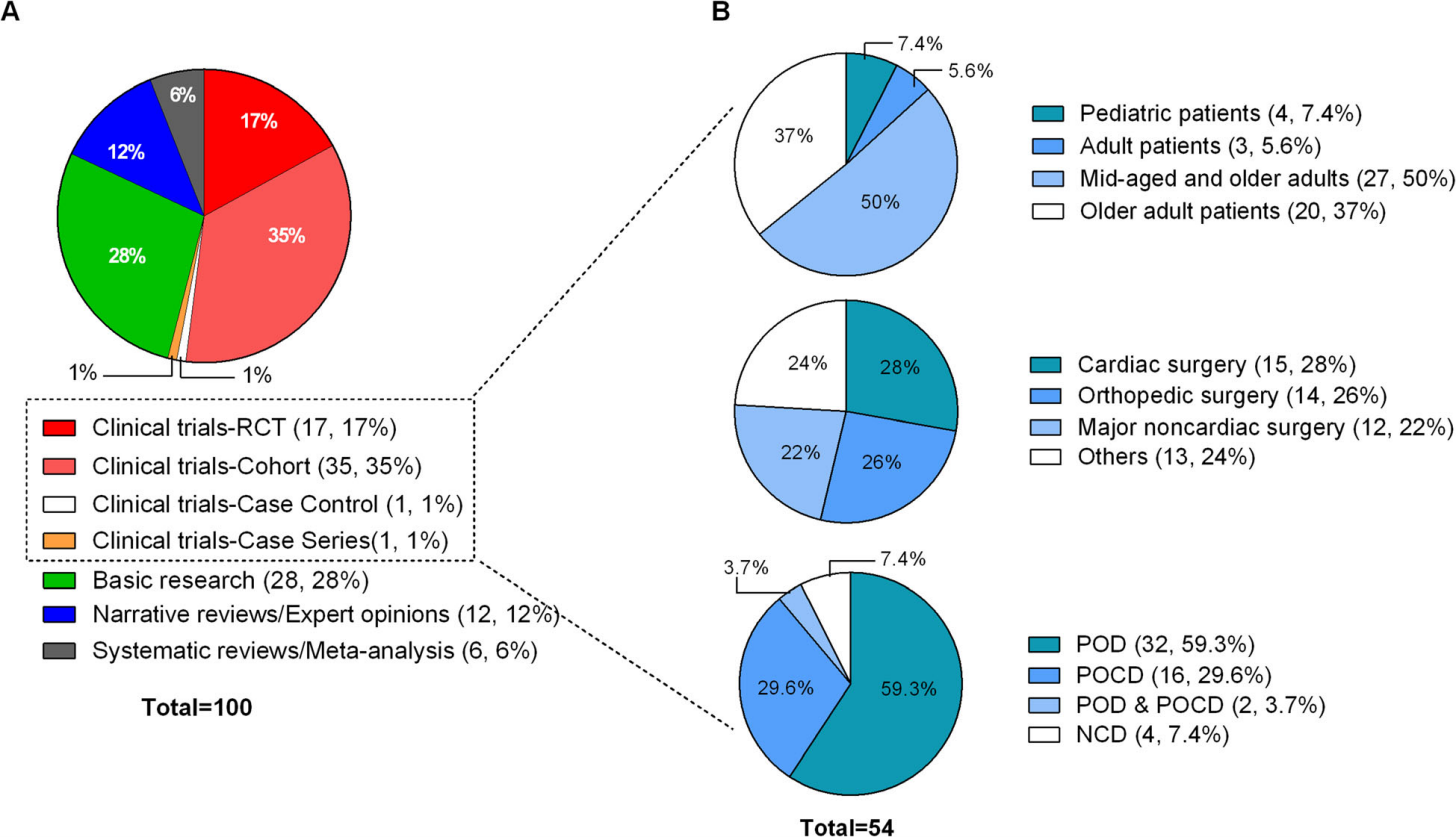
Study designs of the 100 most highly cited papers in PND. PND, perioperative neurocognitive disorder; POD, postoperative delirium; POCD, postoperative cognitive dysfunction; NCD, neurocognitive disorder; RCT, randomized controlled trial

The most common article types were clinical trials (*n* = 54) and experimental studies (*n* = 28); they were followed by narrative reviews (*n* = 12) and systematic reviews or meta-analyses (*n* = 6) (Fig. 2 A). Over half of the high-citation articles were clinical studies (n = 54); thus, we further classified such studies. Half of those studies (27 trials, 50 %) examined mid-aged and older adult patients; 20 studies (37 %) investigated older adult patients. Only four studies (7.4 %) investigated developmental children, and three (5.6 %) examined adults. The type of surgery reported in 54 clinical studies could be divided into four categories: cardiac surgery (15 trials, 28 %); orthopedic surgery (14 trials, 26 %); major non-cardiac surgery (12 trials, 22 %); and other (13 trials, 24 %). With respect to research themes, 32 of 54 trials (59.3 %) investigated POD; 16 trials (29.6 %) examined POCD. The authors of four studies used the term “neurocognitive disorder (NCD)”; two other studies reported on both POD and POCD (Fig. 2B).

### Citations

Overall, the citation count ranged from 111 to 1248 citations; the annual citation rate (ACR), which was number of citations divided by the number of years the article has been published (In this study, the time point was set at December 31st, 2020), ranged from 3.8 to 97.5 citations/year. The top 10 cited papers were shown in Table 1 with eight of them focusing on clinical studies and the other two focusing on basic science. Among the 8 clinical studies, the contents of the studies included the predictors, risk factor and poor outcomes of the POD or POCD. While the 2 basic science studies focused on the neuroinflammatory theory exploring the mechanisms of POCD. By count, the most cited paper was a multicenter, prospective cohort study by Moller et al. published in 1998 and entitled “Long-term postoperative cognitive dysfunction in the elderly ISPOCD1 study”, this *Lancet* article had 1248 citations and an ACR of 56.7 citations/year [4]. The second-most cited paper (cited 1233 times and 64.9 citations/year) was entitled “Longitudinal assessment of neurocognitive function after coronary-artery bypass surgery” by Newman et al., published in 2001 in *New England Journal of Medicine* [5]. The paper with the highest ACR was a randomized controlled clinical trial entitled “Neurodevelopmental outcome at 2 years of age after general anaesthesia and awake-regional anaesthesia in infancy (GAS): an international multicentre, randomised controlled trial” by Davidson et al.; published in 2016 in *Lancet*, it was the seventh-most cited paper [6]. Almost half of the papers (n = 45) were cited more than 200 times.

**Table 1:** Top 10 most cited articles focusing on cognitive change associated with anesthesia and surgery

Rank number	Citations^a^	ACR	Year	First Author	Title	Journal	Country
1	1248	56.7	1998	Moller, JT	Long-term postoperative cognitive dysfunction in the elderly: ISPOCD1 study	Lancet	United States
2	1233	64.9	2001	Newman, MF	Longitudinal assessment of neurocognitive function after coronary-artery bypass surgery	New England Journal of Medicine	United States
3	679	56.6	2008	Monk, TG	Predictors of cognitive dysfunction after major noncardiac surgery	Anesthesiology	United States
4	568	21.8	1994	Marcantonio, ER	A clinical prediction rule for delirium after elective noncardiac surgery.	JAMA	United States
5	451	41.0	2009	Steinmetz, J	Long-term Consequences of Postoperative Cognitive Dysfunction	Anesthesiology	Denmark
6	403	20.2	2000	Marcantonio, ER	Delirium is independently associated with poor functional recovery after hip fracture	Journal of the American Geriatrics Society	United States
7	390	97.5	2016	Davidson, AJ	Neurodevelopmental outcome at 2 years of age after general anaesthesia and awake-regional anaesthesia in infancy (GAS): an international multicentre, randomised controlled trial	Lancet	Australia
8	388	38.8	2010	Cibelli, M	Role of Interleukin-1 beta in Postoperative Cognitive Dysfunction	Annals of Neurology	England
9	371	37.1	2010	Terrando, N	Tumor necrosis factor-alpha triggers a cytokine cascade yielding postoperative cognitive decline	PNAS	United States
10	369	21.7	2003	Morrison, RS	Relationship between pain and opioid analgesics on the development of delirium following hip fracture	The journals of gerontology. Series A, Biological sciences and medical sciences	United States

^a^ the citations times is according to WoS Core; *ACR*, annual citation rate, citation/year

### Journals

The papers were published in 49 journals. The top 10 cited journals and their impact factors in 2019 appear in Table 2. Of the 49 journals, the most frequently cited journal was* Anesthesiology* (n = 17); it was followed by *Anesthesia & Analgesia* (*n* = 9), *Journal of the American Geriatrics Society* (*n* = 6), *Lancet* (*n* = 6), *JAMA* (*n* = 4), and *Journal of Thoracic and Cardiovascular Surgery* (n = 4). Of the top 10 cited journals, seven were American and three were British.

**Table 2:** The top 10 sources contributing to the top 100 publication

Rank number	Journal	No. of top 100 articles	Country of origin	Impact factor 2019
1	Anesthesiology	17	United States	7.067
2	Anesthesia & Analgesia	9	United States	4.305
3	Journal of the American Geriatrics Society	6	United States	4.18
4	Lancet	6	England	60.392
5	JAMA: the journal of the American Medical Association	4	United States	45.54
6	Journal of Thoracic and Cardiovascular Surgery	4	United States	4.451
7	Annals of Neurology	3	United States	9.037
8	British Journal of Anaesthesia	3	England	6.88
9	Acta Anaesthesiologica Scandinavica	3	England	2.05
10	Psychosomatics	3	United States	2.00

### Authors

The top 10 authors published most papers for PND and the number of citations appear in Table 3. The author with the highest number of top-100 papers was Marcantonio, with eight papers (first author in four of them). Marcantonio was followed by Xie, with seven papers (five as the last author). In joint third place were Maze, Rasmussen, Grosby, and Culley: they each had six papers. Maze was the last author in five of the six papers. Rasmussen’s papers received the highest number of total citations (2668). In the fourth place were Moller, Hanning, and Dong; they each had five papers. Both Moller and Hanning obtained a considerable number of citations (2217 each). Their citation number followed that of Marcantonio (2362). In fifth place, Ma had four papers, and he was the last author in one of them.

**Table 3:** Authors with top-10 number of papers included in the 100 most-cited

Rank number	Name	Total Publications	First Author	Co-Author	Last Author	Total Citations	Mean Citations/paper
1	Marcantonio ER	8	4	1	3	2362	295
2	Xie ZC	7	1	1	5	1067	152
3	Maze M	6	0	1	5	1787	298
4	Rasmussen LS	6	1	4	1	2668	445
5	Crosby G	6	0	4	2	1004	167
6	Culley DJ	6	2	4	0	1004	167
7	Moller JT	5	1	0	4	2217	443
8	Hanning CD	5	0	5	0	2217	443
9	Dong YL	5	0	5	0	776	155
10	Ma DQ	4	0	3	1	1218	305

### Newest versus older papers

To evaluate changes in PND research areas with time, we divided the top 100 cited papers into the 50 older and 50 newest papers as mentioned in Search Strategy part (Table 4). Compared with older papers, newest papers had statistically higher ACRs (24.9 versus 15.3 citations/year; *P* = 0.001), more funding disclosures (76 % versus 46 %; *P* = 0.002), more open access publications (62 % versus 18 %; *P* < 0.001), and different article focus (*P* < 0.05). Compared with older papers, newest papers focused more on basic science outcomes (40 % versus 16 %), less on clinical outcomes (46 % versus 62 %), and there were fewer review articles (including expert opinions and meta-analysis articles; 14 % versus 22 %). We observed no significant differences in the total number of citations, number of authors, the involvement of multiple institutions, and research areas (all *P* > 0.05).

**Table 4:** Comparison of papers published between older (before February 2008) and newest ones (after June 2008)

Parameter	Older group (*n* = 50)	Newest group (*n* = 50)	*P* value
Quantitative (mean, range)			
Total Citations, number	268.6 (122–1248)	201.6 (111–451)	0.055
Citations/year, number	15.3 (3.8–64.9)	24.9 (10.3–97.5)	**0.001**
Author, number	6.9 (1–20)	8.1 (1–20)	0.190
Qualitative (n, %)			
Multiple institutions	28 (46 %)	35 (70 %)	0.147
Article focus			**0.027**
Clinical outcomes	31 (62 %)	23 (46 %)	
Basic science outcomes	8 (16 %)	20 (40 %)	
Review article^a^	11(22 %)	7 (14 %)	
Research areas			0.086
Anesthesiology	19 (38 %)	18 (36 %)	
General & Internal. Medicine	11 (22 %)	8 (16 %)	
Geriatrics & Gerontology	9 (18 %)	5 (10 %)	
Neurosciences & Neurology	2 (4 %)	10 (20 %)	
Other topics	9 (18 %)	9 (18 %)	
Funding disclosed	23(46 %)	38(76 %)	**0.002**
National/institutional	23/23 (100 %)	36/38 (95 %)	
Industry	0/23 (0 %)	2/38 (5 %)	
Open access^b^	9 (18 %)	31 (62 %)	**<0.001**

The publication year of the top 100 papers was range 1993 to 2016, the median year of publication was 2008. The top 100 papers were divided into 50 older papers (before February 2008) and 50 newest ones (after June 2008) according to the publication date order. Bold type indicated statistical significance. ^a^, Both of expert opinions and meta-analysis articles are regarded as review articles here. ^b^ Open access journals are available for free public access.

### Citations per year

**Fig. 3:**
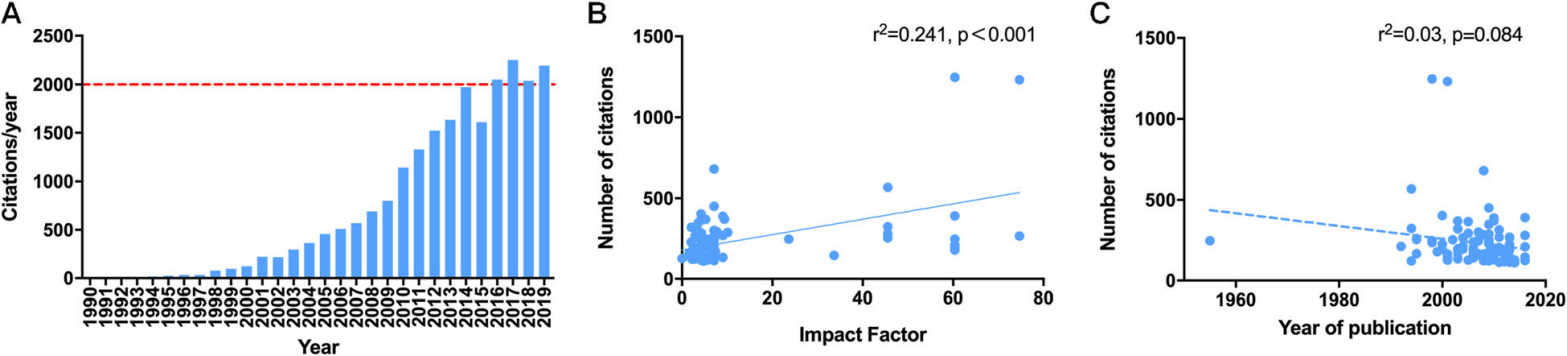
*** A***, The total cited frequencies each year of all articles in the top 100. ***B***, Linear correlation between impact factors of journals where papers were published and number of citations of articles included in the top 100 list. ***C***, Linear correlation between year of publication of articles in the top 100 list and their number of citations

The total number of annual citations among the journals showed a steady increase from 1990 to 2019. From 2016 to 2019, the total number of citations per year exceeded 2000 (Fig. 3 A).

### Correlation analysis

The impact factors of the journals were positively correlated with the number of cited articles (r = 0.491, *P* < 0.001; Fig. 3B). However, the number of citations showed no correlation with the year of publication (*r* = − 0.174, *P* > 0.05; Fig. 3 C).

## Discussion

In this study, we identified the top 100 cited PND papers and analyzed their nature, content, and changes with time. The results of our bibliometric analysis indicated that among the papers, clinical trials accounted for over half; the papers focused mainly on mid-aged and older adult patients; the research theme was largely cardiac surgery and POD. As evident by citation frequency, the nature of high-impact studies has changed with time. The changes included that the later the publication year, the higher the citation rates, greater funding disclosures, more focus on basic research, and more open access publications; those trends indicate the possible future direction of research in this field.

Among the 100 most cited papers, the publication year ranged from 1955 to 2016. The number of published papers showed a steady growth, reflecting the progressive development of research into PND. The greatest number of citations occurred in the 2000 s, indicating that this research area gained considerable attention in that decade. Expansion in the number of PND studies may be associated with the multicenter, prospective cohort ISPOCD1 study published by Moller et al. in 1998 [4]; which was the most cited paper in PND, and it had tremendous historical significance. Other groundbreaking works had a similar impact. One was the earliest paper to appear on our list when Bedford reported the occurrence of dementia in older adults following operations under general anesthesia in 1955 [2]. It was a milestone paper in PND research, highlighting cerebral complications during the perioperative period. Another paper, dating from 2018, examined the nomenclature of cognitive changes associated with anesthesia and surgery. That paper recommended using the term “PND” as an overarching concept to describe preoperative or postoperative cognitive impairment; although not included in the top 100 list, it led to PND becoming listed as a neurocognitive diagnosis in Diagnostic and Statistical Manual of Mental Disorders-5 [1], showing an important influence on the PND research and clinical practice.

In our analysis, we identified the United States as the leading country in total number of publications, followed by England. Among the top 10 journals with the highest number of publications (58 published papers), seven were in the United States (46 published papers), and the other three journals were in England (12 published papers). The authors of the top 100 cited papers also showed a relatively concentrated pattern. The top 10 most highly cited authors of the 100 most cited papers had 58 publications, contributing more than half.

Among the top 100 cited papers, 54 were clinical trials; of those, 50 % investigated mid-aged and older adult patients and 37 % examined older adult patients. This finding may be related to aging of the global population and vulnerability of older adults to postoperative cognitive impairment. Interestingly, 7.4 % of the papers focused on developmental children. The GAS study published in *Lancet* in 2016 [6] had 390 citations; it ranked seventh among the 10 most cited papers and had the highest ACR (97.5 citations/year). This result suggests that the influence of anesthesia on children’s neurological development is a key research area in PND. The pediatric anesthesia neurodevelopment assessment (PANDA) study was another highly cited paper, focusing on young children aged under 36 months and published in *JAMA* [7]. It had the second-highest ACR (69.25 citations/year), but it was not included in the top 10 cited papers because of its publication year of 2016.

Among the 54 clinical trials, 28 % investigated patients who underwent cardiac surgery; 26 % examined orthopedic surgery and 22 % chose non-cardiac surgery. This finding is consistent with the view that major surgery constitutes a risk factor for PND [8]. Among the clinical trials, 59.3 % focused on POD and 29.6 % on POCD. That result may be related to differences in the trials with respect to onset period, incidence, and diagnostic criteria. POD is an acute event, comprising a set of fluctuating changes in attention, mental status, and level of consciousness; it reportedly occurs in 10–60 % of older surgical patients, varying by surgical procedure, such as if it is a major or minor surgery, the use of extracorporeal circulation, and the hypotension during the procedure [9]. POCD is cognitive decline diagnosed up to 30 days after a procedure; its incidence is approximately 10–12 % [10]. The scales of Confusion Assessment Method (CAM) or the CAM adapted for the intensive care unit are mostly used to diagnose POD; POCD diagnosis requires more complex tests [11]. With its relatively acute course, higher incidence, and simpler diagnosis, POD is more often focused as the primary outcome in clinical trials.

When comparing newest with older articles, we found that newest papers had higher ACRs. This finding indicates that PND received considerable attention among researchers and that cognitive function drew increased interest in perioperative medicine. This may be partly because that researchers threw light on the risk factors and preventions of PND, and the more adequate monitor devices such as the use of electroencephalogram (EEG) monitors. A research in 2013 indicated the brain function monitoring using the bispectral index (BIS) decreased the risk of POCD at 3 months after surgery, accordingly [12]. The greater number of funding disclosures reflects the governmental support for this area of research. As the global population aging, PND has become a great medical challenge. The governmental and funding support also influence the development direction of a research field to some extent. The greater open access publications increased the impact of findings. The stronger focus on basic science outcomes in newest papers indicates that the main area of PND research changed from clinical phenomena to underlying mechanisms, expecting to have better prevention or therapy for PND. This result also suggests future PND research directions.

Our bibliometric analysis has inherent limitations. First, some newly published, high-quality papers were unable to gain sufficient citations to appear among the top 100 [13]. Therefore, in this study, the papers published in recent years (from 2016 to 2020) were not included in the top 100, but it does not mean those papers are not that important. Second, papers published in journals with higher impact factors may gain greater attention and thus have more citations [14]. To evaluate any inherent bias, we analyzed the correlation between the number of citations of articles in our list and the impact factors of journals where the papers were published; we also examined the correlation between the number of citations and year of publication. We observed a positive correlation between the number of citations and the journals’ impact factors, which may be reflective of the inherent bias of high impact factors; we found no correlation between the number of citations and year of publication in the current study.

## Conclusions

We compiled a comprehensive list of the 100 most cited papers dealing with PND to examine the current status and global trends in PND research. Despite its limitations, our bibliometric analysis found a steadily growing focus on PND. Compared with older papers, newest papers have higher ACRs, more funding disclosures, more open access publications, and greater focus on basic science. High-quality clinical trials with a stronger journal impact factor appear to receive more citations; however, basic science studies may increase as a future area of research in the pathogenesis of PND.

## Data Availability

All data and related metadata underlying the findings reported in our study are provided as part of the submitted article. Additional data is available on reasonable request from the corresponding author.

## Abbreviations

PND: Perioperative neurocognitive disorder
POD: Postoperative delirium
POCD: Postoperative cognitive dysfunction
ACR: Annual citation rate
